# Inhibition of ACOX1 enhances the therapeutic efficacy of obeticholic acid in treating non-alcoholic fatty liver disease and mitigates its lipotoxicity

**DOI:** 10.3389/fphar.2024.1366479

**Published:** 2024-03-26

**Authors:** Yuping Yang, Weinan Yuan, Kun He, Chuangzhen Lin, Shenshen Du, Yanqi Kou, Biao Nie

**Affiliations:** ^1^ Department of Gastroenterology, Affiliated Hospital of Guangdong Medical University, Guangdong Medical University, Zhanjiang, Guangdong, China; ^2^ Department of Gastroenterology, The First Affiliated Hospital of Jinan University, Jinan University, Guangzhou, Guangdong, China; ^3^ Department of Gastroenterology, Inflammatory Bowel Diseases Research Center, The Third Affiliated Hospital of Guangzhou Medical University, Guangzhou, Guangdong, China

**Keywords:** non-alcoholic fatty liver disease, obeticholic acid, farnesoid X receptor, ACOX1, lipotoxicity

## Abstract

**Background and aims:**

High-dose Obeticholic acid exhibits promise for non-alcoholic fatty liver disease (NAFLD) treatment but can induce lipotoxicity. Our study sought to understand this mechanism and propose a solution.

**Approach and Results:**

In a non-alcoholic fatty liver disease (NAFLD) model induced by a high-fat diet in FXR^−/−^ mice, we pinpointed that FXR regulated the expression of ACOX1 through RNA-Seq analysis. In the livers of FXR^−/−^ mice, both ACOX1 mRNA and protein expression notably decreased. In both HL-7702 and HEP-G2 cells, the silencing of FXR through shRNA plasmids decreased ACOX1 expression, while FXR activation with GW4064 increased it. These effects were reversible with the ACOX1-specific inhibitor, 10,12-Tricosadiynoic acid. In the NAFLD model of FXR^−/−^ mice, The activation of ACOX1 is correlated with elevated serum LDL, triglycerides, and aggravated hepatic steatosis. However, the combination of 10,12-Tricosadiynoic acid with low-dose obeticholic acid effectively treated hepatic steatosis, reducing LDL levels in the NAFLD model of wild-type mice. This combination therapy demonstrated efficacy comparable to high-dose obeticholic acid alone. Notably, the combined drug regimen treats hepatic steatosis by inhibiting the IL-1β and α-SMA pathways in NAFLD.

**Conclusion:**

Combining ACOX1-specific inhibitors with low-dose obeticholic acid effectively treats high-fat diet-induced hepatic steatosis and reduces serum LDL. This approach enhances the therapeutic effects of obeticholic acid and mitigates its lipotoxicity by inhibiting the IL-1β and α-SMA pathways.

## 1 Introduction

Non-alcoholic fatty liver disease (NAFLD), also known as metabolic dysfunction-associated fatty liver disease (MASLD). MASLD is a liver condition characterized by fat accumulation, linked to disruptions in metabolic function. MASLD often involves hepatic steatosis and is associated with factors such as obesity, insulin resistance, and abnormal glucose metabolism (Eslam et al., 2020). According to the latest data, the global prevalence of NAFLD is 30.05%, with the highest prevalence in Latin America at 44.37% and the lowest prevalence in Western Europe at 25.10% (Younossi et al., 2023). According to a meta-analysis, the prevalence of NAFLD in overweight individuals was 69.99%, while the prevalence of non-alcoholic steatohepatitis (NASH) was 33.5% (Quek et al., 2023). NAFLD has become one of the leading causes of liver transplantation. However, there are no effective drugs approved by the U.S. Food and Drug Administration or the European Union to halt the progression of NAFLD, posing a significant threat to public health (Paternostro and Trauner, 2022).

Farnesoid X receptor (FXR) agonists appear to be the most promising drugs for NAFLD. The FDA and the EU recently approved obeticholic acid (OCA), a potent FXR agonist, to treat primary biliary cholangitis (Nevens et al., 2016). OCA is the only drug that has completed a Phase III clinical study for treating NAFLD (Younossi et al., 2019). A high dose (25 mg) of OCA significantly improved fibrosis and NASH disease activity scores in patients with NASH (Younossi et al., 2019). However, this dose of OCA caused pruritus, elevated LDL, and decreased HDL. Dose-dependent pruritus may lead to treatment discontinuation in some patients, while lipotoxicity may increase the risk of cardiovascular disease. Due to these side effects, OCA is not recommended for treating NAFLD (Neuschwander-Tetri et al., 2015; Younossi et al., 2019; Chapman and Lynch, 2020). OCA is well-tolerated at regular or low doses but is not as effective in treating NASH (Younossi et al., 2019). According to our preliminary study, the side effects of OCA depend on the activation and presence of FXR (Lin et al., 2022a). Our previous study found that OCA can alleviate NAFLD by reducing hepatic fatty acid uptake through the inhibition of fatty acid transporter protein 5, and this pathway is independent of FXR (Lin et al., 2022b). Our present study suggested that ACOX1 is a potential gene for adverse effects due to FXR activation in NAFLD. Therefore, targeting ACOX1 along with OCA could be a promising strategy for treating NAFLD. However, further studies are needed to confirm the efficacy and safety of this combination therapy.

Acyl-CoA oxidase 1 (ACOX1) is a critical enzyme in the β-oxidation of peroxisomal very-long-chain fatty acids. The ACOX1 gene can translate into two proteins with different molecular weights, namely 71 kDa and 22 kDa. The main functional protein is the 71 kDa variant. It catalyzes the desaturation of acyl-CoAs to 2-trans-enoyl-CoAs, producing two molecules of acetyl-CoA. This pathway-derived acetyl-CoA promotes lipid autophagy and inhibits mitochondrial fatty acid β-oxidation, contributing to the onset and progression of NAFLD (Mannaerts et al., 2000; Ding et al., 2021). In contrast, liver-specific ACOX1 knockout mice exhibited normal growth and reproduction. Moreover, liver-specific knockout of the ACOX1 gene reduced serum LDL and triglyceride levels and decreased hepatic steatosis in mice (He et al., 2020a). 10,12-Tricosadiynoic acid, an ACOX1-specific inhibitor, improves hepatic steatosis in rats (Zeng et al., 2017). The role of ACOX1 in steatohepatitis remains unclear. Whether FXR induces lipotoxicity by activating ACOX1 remains unknown. Further research is needed to clarify this relationship. Additionally, the efficacy of ACOX1-targeted drugs combined with OCA for treating NAFLD is not yet known and requires further investigation.

Using *in vitro* and *in vivo* experiments, we aimed to confirm that ACOX1 causes the side effects of FXR activation. Additionally, we would explore the efficacy and the mechanism of ACOX1-targeted drugs combined with OCA in treating NAFLD.

## 2 Materials and methods

### 2.1 Cell experiments

In this experiment, HL-7702 (Zrbiorise, BIOCC2225h054) and HEP-G2 cells (Procell, CL-0594) were transfected with FXR ShRNA plasmids (Sequence of ShRNA plasmids was showed in Supplementary Table S1) or a vector expression construct to establish stable cell lines. The vector used for shRNA plasmids is “pSLenti-U6-shRNA-CMV-EGFP-F2A-Puro-WPRE.” The vector structure is shown in Supplementary Figure S1. The transfection was performed using a Lipofectamine^TM^ 3000 kit according to the manufacturer’s instructions (Invitrogen™, L3000015). After 48 h of transfection, the total cellular RNA and protein were extracted from the transfected cells for Real-Time Quantitative PCR and Western blotting, respectively.

Induction experiments were also conducted *in vitro* on HL-7702 and HEP-G2 cells. The cells were treated with 65 nM and 500 nM concentrations of GW4064 (Meryer, 278,779-30-9) and 10,12-Tricosadiynoic acid (Macklin, 10,12-Tricosadiynoic acid), respectively. After 48 h of drug incubation, the cells were harvested to extract total RNA and protein for Real-Time Quantitative PCR and Western blotting, respectively.

### 2.2 RNA sequencing (RNA-Seq) analysis

The sequencing of single-end libraries with the BGISEQ-500 platform was used to construct a complementary DNA (cDNA) library from total RNA extracted from the liver. The alignment of the resulting reads was performed using Bowtie2 software (version 2.2.5) and gene expression levels were quantified using RSEM (version 1.2.8). Differential gene expression and significance based on read counts were calculated using DESeq2. Differentially expressed genes between the FXR^−/−^ mice group and the wild-type mice group, both induced by a high-fat diet for 14 weeks, were identified as having a fold change greater than 2 and a Q-value (adjusted *p*-value) less than 0.05.

### 2.3 Animal experiments

All experimental mice in this study were of the C57BL/6N strain background. Eight-week-old male mice were used for experiments. Mice were housed in a pathogen‐free animal facility on a 12‐h dark/light cycle and had free access to water and food. The generation of whole-body FXR knockout (FXR^−/−^) mice was previously described in studies conducted by the research team (Lin et al., 2022a; Lin et al., 2022b).

The mice were fed a high-fat diet (60% kcal from fat, D12492, Research Diets) for 14 weeks to induce a NAFLD model. The therapeutic group was given the corresponding drug intervention for 6 weeks, starting from the 9th week of the high-fat diet. The control group received a placebo or the corresponding solvent.

WY-14643 (an indirect ACOX1 agonists: WY14643 is an agonist of PPAR alpha, indirectly activating ACOX1 by stimulating PPAR alpha15,16; Selleck, WY-14643) was added to the liquid diets at a concentration of 10 mg/L as previously described 17. In review Obeticholic acid (Bidepharm, BD222251) was administered orally by gavage at different dosages, including 160 mg/kg for the high dose, 40 mg/kg for the normal dose, 10 mg/kg for the middle dose, and 2.5 mg/kg for the low dose. 10,12-Tricosadiynoic acid (Macklin, T867980) was administered orally by gavage at 224 μg/kg per day14.

All animals received human care, and the study protocols complied with the institution’s guidelines, and the Laboratory Animal Ethics Committee of Jinan University approved the animal experiments. (Approval No: IACUC-20221010-12 and IACUC-20221010-13).

The scoring for Nonalcoholic Steatohepatitis (NASH) and fibrosis stage utilizes the internationally recognized “Histological Scoring System for Nonalcoholic Fatty Liver Disease” (Nonalcoholic Steatohepatitis Clinical Research Network, https://tpis.upmc.com/changebody.cfm?url=/tpis/schema/nafld2006.jsp#Total) (Kleiner et al., 2005). The NASH score comprises three indicators: Steatosis, Lobular Inflammation, and Hepatocyte Ballooning, with a score of 5-8 indicating a diagnosis of NASH.

### 2.4 Metabolic plasma analysis

This is a standard procedure to measure biochemical markers in mice. After an overnight fast, blood samples are collected. The levels of various markers including alanine aminotransferase (ALT), aspartate aminotransferase (AST), total bilirubin(TBIL), direct Bilirubin (DBIL), Albumin (ALB), total cholesterol (CHO), triglyceride (TG),high-density lipoprotein (HDL), low-density lipoprotein (LDL), glucose (GLU), γ-glutamyltransferase (r-GT) and total bile acid (TBA) are measured using a HITACHI 7180 automated biochemical analyzer (Gokita et al., 2020). The manufacturer’s instructions for the respective kits (Biotechnology) are followed for each test. The instructions and detailed information for all kits are on the official website (http://www.gz-donglin.cn/jiageku.asp?sortId=127&id=954). The detailed information for each kit can be found in Supplementary Table S2.

### 2.5 Western blotting analysis

In equal amounts, the denatured protein was separated by a 10% SDS-PAGE gel and subsequently transferred onto a PVDF membrane (Millipore, United States). Following blocking, the membranes were incubated overnight with specific primary antibodies, including anti-ACOX1 (Proteintech, 10957-1-AP), anti-α-SMA (Proteintech, 14395-1-AP), anti-IL-1β (Abcam, ab18955), or anti-GAPDH (Proteintech, 60,004-1-lg). Afterward, horseradish peroxidase-conjugated antibodies were applied, and protein bands were visualized using a chemiluminescence kit (Millipore, United States) and the LAS Chemiluminescent Imaging System (LAS500, United States). Quantification of the bands was performed using ImageJ.

### 2.6 Real-time quantitative PCR

The standard procedure for gene expression analysis involves RNA extraction using TRIzol reagent, followed by reverse transcription of RNA into cDNA using a cDNA synthesis kit (Accurate Biotechnology, AG11728). cDNA synthesis steps: The reaction mixture, excluding genomic DNA, was incubated at 42°C for 2 min, followed by cooling to 4°C for 3 min. Subsequently, the cDNA synthesis reaction mixture was added, and the incubation was carried out at 37°C for 15 min, followed by 85°C for 5 s, and a final step at 4°C for 3 min (The resulting product was stored at- 20°C.) Real-time quantitative PCR (qPCR) is performed in a CFX96 Real-Time PCR Detection System to measure the expression levels of target genes using specific primers. The RT-qPCR kits (Accurate Biotechnology, AG11732) contain all the necessary reagents and probes for the reaction. The results are analyzed to determine the relative expression levels of the target genes in different samples. The primers used in the study are listed in Supplementary Table S3.

### 2.7 Statistical analysis

The mean ± standard error of the mean was used to display the data. Two-tailed Student’s t-test was used to compare two groups and one-way ANOVA with Tukey *post hoc* analysis was used to compare multiple groups. The significance level was set at *p* < 0.05. GraphPad Prism9.4.1 was used for data analysis.

## 3 Results

### 3.1 ACOX1 was identified as a downstream gene driven by FXR in liver RNA-seq

The present study performed RNA sequencing on liver samples from wild-type mice and FXR^−/−^ mice after 14 weeks of feeding a high-fat diet. Of the 15,988 genes expressed in both groups, 1451 were upregulated and 2035 were downregulated in the FXR^−/−^ mice group (Supplementary Figure S2A, B) (*p* < 0.05). KEGG pathway analysis revealed that 142 differentially expressed genes were related to lipid metabolism in FXR^−/−^ mice (Supplementary Figure S2C). Since obeticholic acid’s adverse effects are mainly lipotoxic, we focused on differentially expressed genes associated with lipid metabolism. It appears that the differential genes identified through KEGG module analysis in this study are primarily involved in various aspects of lipid metabolism, including beta-oxidation, triacylglycerol biosynthesis, beta-oxidation of peroxisomes (VLCFA), fatty acid elongation in the reticulum and mitochondria, bile acid biosynthesis and fatty acid biosynthesis and elongation (Supplementary Figure S2D). Among the 142 differential genes, ACOX1 was identified as the primary driver gene in Gene Mutual Network analysis and KEGG network analysis showed that the genes directly associated with ACOX1 were mainly related to fatty acid metabolism or degradation, biosynthesis of unsaturated fatty acids and the PPAR signaling pathway (Supplementary Figure S2E, F, respectively). Furthermore, the mRNA expression of ACOX1 was downregulated in FXR^−/−^ mice (Supplementary Figure S2G, H, respectively) (*p* < 0.05). Thus, ACOX1 is identified as a downstream gene driven by FXR in liver RNA-seq.

### 3.2 FXR regulated ACOX1 mRNA and protein expression in the cell line

**FIGURE 1:**
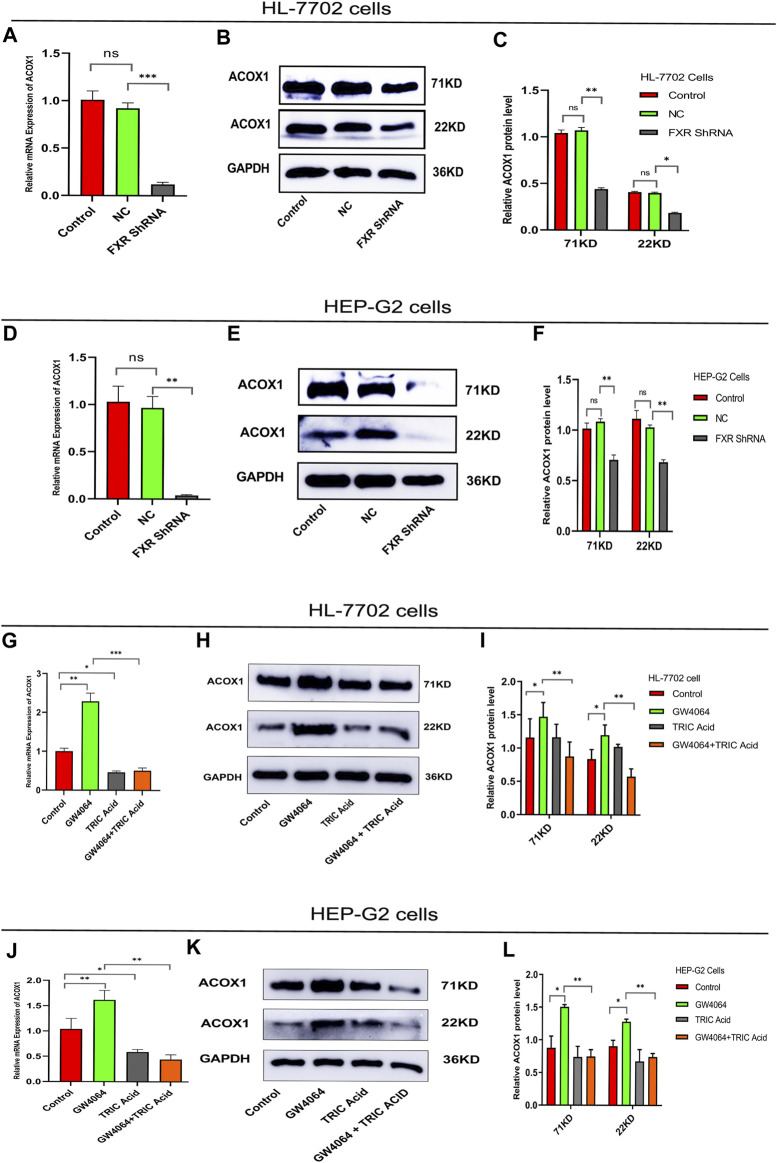
FXR regulates ACOX1 expression. **(A–F)** shRNA plasmid-mediated silencing of the FXR gene decreased ACOX1 expression in HL-7702 and HEP-G2 cells, respectively. **(G–L)** FXR activation with GW4064 increased ACOX1 expression; this effect was reversed by 10,12-Tricosadiynoic acid in HL-7702 and HEP-G2 cells, respectively. Control: Blank control group; NC: Negative control group; FXR ShRNA: ShRNA plasmid silences FXR gene group; TRIC Acid: 10,12-tricosadiynoic acid). * indicates *p*-value <0.05; ** indicates *p*-value <0.01; *** indicates *p*-value <0.001; **** indicates *p*-value <0.0001; ns indicates *p*-value >0.05.

To investigate the relationship between FXR and ACOX1,a plasmid vector containing the RNA-Short hairpin of FXR (FXR shRNA)was constructed. FXR shRNA plasmids were used to silence FXR gene expression in HL-7702 and HEP-G2 cells, respectively. These led to a decrease in ACOX1 mRNA and protein expression (Figures 1A–F) (*p* < 0.05). On the other hand, activating FXR with GW4064 increased ACOX1 mRNA and protein expression in these cell lines (Figures 1G–L) (*p* < 0.05). The induction of ACOX1 expression by FXR activation was reversed by using 10,12-Tricosadiynoic acid, an ACOX1-specific inhibitor (Figures 1G–L) (*p* < 0.05). These results suggest that FXR regulates ACOX1 mRNA and protein expression.

### 3.3 The activation of ACOX1 is correlated with the worsening of steatohepatitis and an increase in LDL levels in FXR^−/−^ mice

**FIGURE 2:**
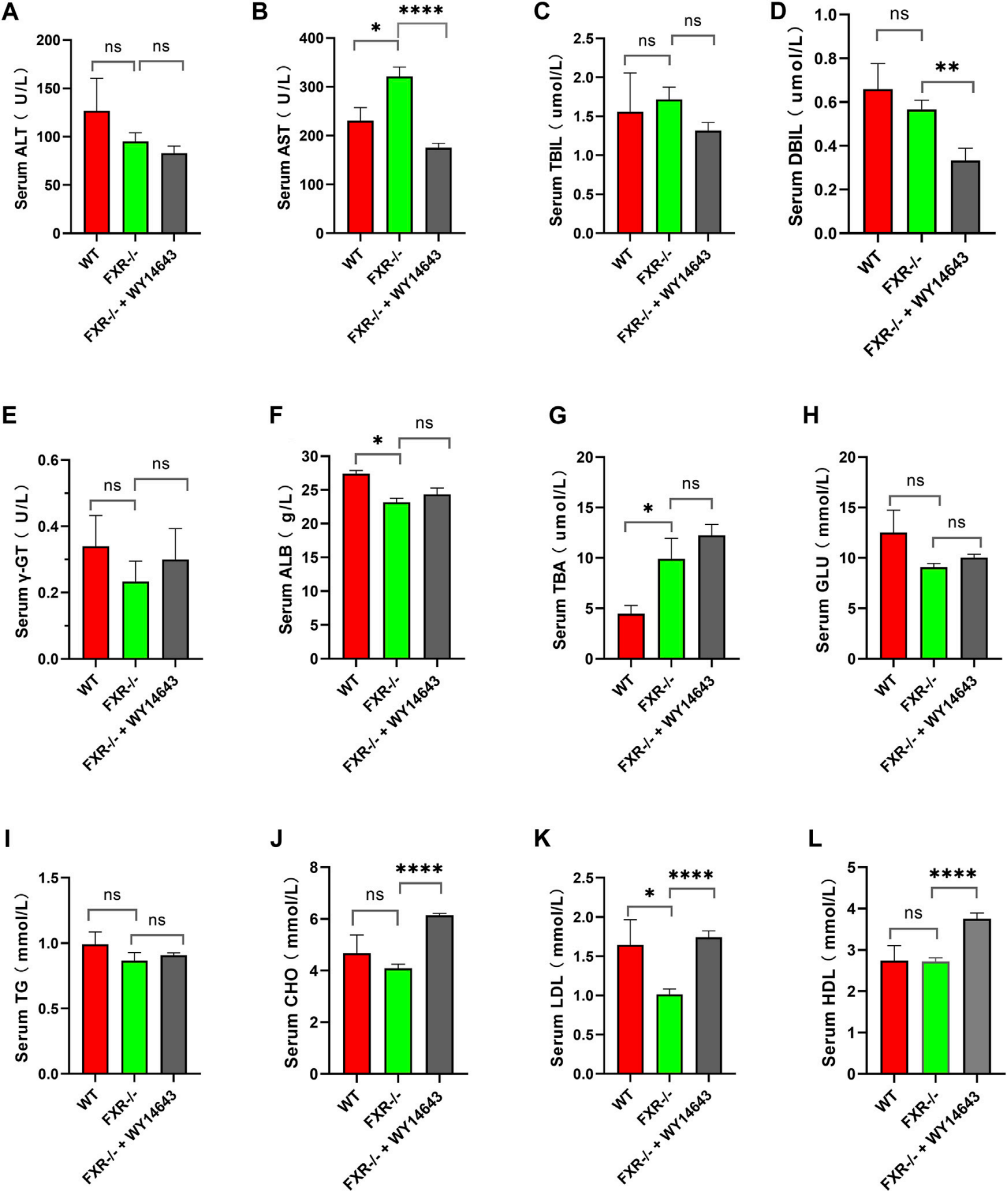
The activation of ACOX1 is correlated with the elevation of serum LDL levels in FXR^−/−^ mice. (To induce nonalcoholic fatty liver disease, wild-type mice and FXR^−/−^ mice were fed high-fat diets for 14 weeks. Each group comprises six mice, and there are no significant differences in age and body weight among the groups. **(A–L)** The results of serum ALT, AST, TBIL, DBIL, γ-GT, GLU, TBA, ALB, CHO, TG, LDL, and HDL in three groups, respectively. ALT: alanine aminotransferase; AST: aspartate aminotransferase; TBIL: total bilirubin; DBIL: direct Bilirubin; ALB: Albumin; CHO: total cholesterol; TG: triglyceride; HDL: high-density lipoprotein; LDL: low-density lipoprotein; GLU: glucose, r-GT: γ-glutamyltransferase; TBA: total bile acid. WT: Wild-type mice; FXR^−/−^: FXR gene knockout mice; WY14643: an indirect ACOX1 agonists. * indicates *p*-value <0.05; ** indicates *p*-value <0.01; *** indicates *p*-value <0.001; **** indicates *p*-value <0.0001; ns indicates *p*-value >0.05.

Mice were fed a high-fat diet for 14 weeks to induce NASH. Compared with the wild-type mice control group, serum AST and TBA were increased, and ALB and LDL were decreased in the FXR^−/−^ mice group (Figures 2B, F, G, K, respectively) (*p* < 0.05). Compared to the FXR^−/−^ mice control group, the FXR^−/−^ mice group supplemented with WY14643 showed a decrease in serum AST and DBIL levels and an increase in CHO and LDL levels. There were no significant differences in serum TBIL, γ-GT, GLU, TG, and HDL between the two groups of mice (Figures 2C, E, H, I, L respectively). This suggests a correlation between ACOX1 activation and elevated serum LDL levels in FXR^−/−^ mice (Figures 2B, D, J, K) (*p* < 0.01). These results suggest a correlation between ACOX1 activation and the increase in LDL and liver injury induced by OCA.

**FIGURE 3:**
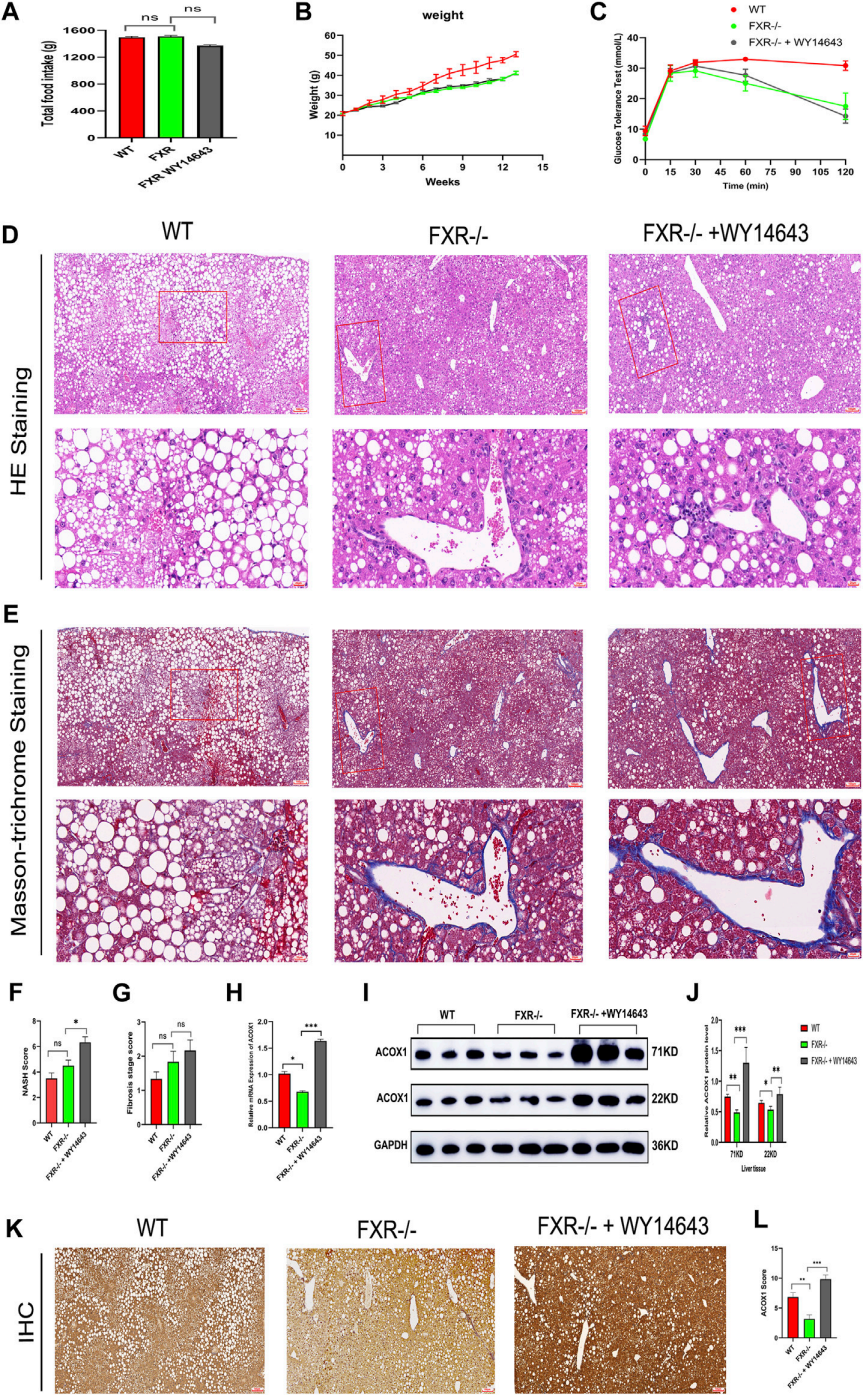
The activation of ACOX1 is correlated with the worsening of steatohepatitis in FXR^−/−^ mice. (To induce nonalcoholic fatty liver disease, wild-type mice and FXR^−/−^ mice were fed high-fat diets for 14 weeks. Each group comprises six mice, and there are no significant differences in age and body weight among the groups. **(A–C)** The results of total food intake, weight gain and glucose tolerance in three groups, respectively. **(D, E)** The results of Hematoxylin-eosin staining and Masson’s trichrome staining in three groups, respectively. **(F,G)** The results of NASH score and fibrosis stage scores in three groups, respectively. **(H–J)** The qPCR and Western Blot results about ACOX1 expression in three groups, respectively. E1-E2: The results of immunohistochemistry staining about ACOX1 expression in three groups.) **(K–L)**: The results of immunohistochemical staining of ACOX1 protein in liver tissue from three groups of mice. WT: Wild-type mice; FXR^−/−^: FXR gene knockout mice; WY14643: an indirect ACOX1 agonists. * indicates *p*-value <0.05; ** indicates *p*-value <0.01; *** indicates *p*-value <0.001; **** indicates *p*-value <0.0001; ns indicates *p*-value >0.05. The images in Figures 3D, E at 400x magnification correspond to the field of view outlined in the red box at 100x magnification.

Three groups of mice had similar food intake, but the wild-type mice group gained weight faster and had impaired glucose tolerance compared to all FXR^−/−^ mice groups (Figures 3A–C). The hematoxylin-eosin staining of liver samples showed that wild-type mice had predominantly macrovesicular steatosis, while FXR^−/−^ mice had predominantly microvesicular steatosis (Figure 3D). The study revealed an increase in inflammatory cell infiltration and hepatic fine balloon-like degeneration in the FXR^−/−^ mice group supplemented with WY14643, with the highest NASH score indicating the worst steatohepatitis (Figure 3F). The results of Masson trichrome staining revealed more severe liver fibrosis in the FXR^−/−^ mouse group supplemented with WY14643, although the difference in liver fibrosis scores did not reach statistical significance (Figures 3E, G). ACOX1 mRNA and protein expression were significantly decreased in the FXR^−/−^ mice control group and increased dramatically in the FXR^−/−^ mice group supplemented with WY14643 (Figures 3H–J) (*p* < 0.05).

Additionally, immunohistochemical staining showed decreased ACOX1 protein expression in the FXR^−/−^ mice control group and significantly increased ACOX1 protein expression in the FXR^−/−^ mice group supplemented with WY14643 (Figures 3K, L) (*p* < 0.05). These results indicate a correlation between ACOX1 activation and the exacerbation of steatohepatitis and an increase in LDL levels in FXR^−/−^ mice.

### 3.4 ACOX1-specific inhibitors combined with middle- or low-dose obeticholic acid significantly improved transaminases, LDL and steatohepatitis in mice

**FIGURE 4:**
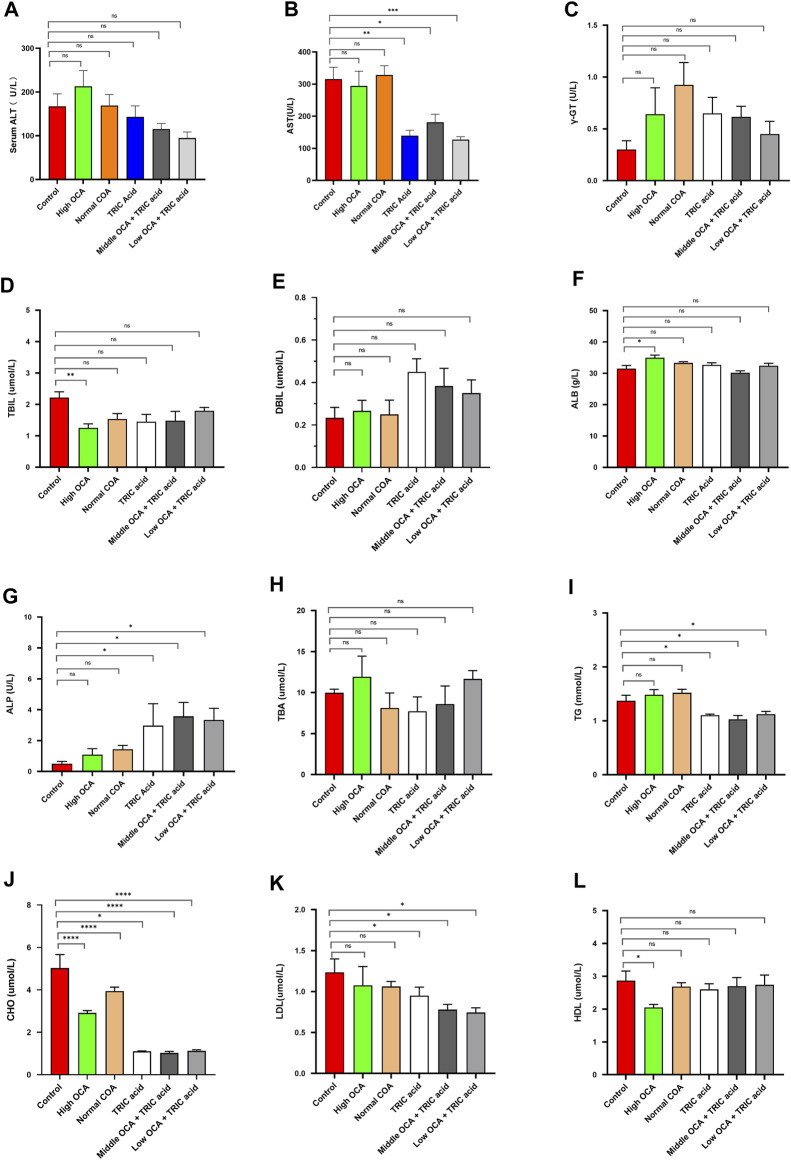
10,12-tricosadiynoic acid combined with middle- or low-dose obeticholic acid significantly improved transaminases and LDL in wild-type mice. (Wild-type mice were fed high-fat diets for 14 weeks to induce nonalcoholic fatty liver disease. Each group comprises six mice, and there are no significant differences in age and body weight among the groups. **(A–L)** The results of serum ALT, AST, TBIL, DBIL, γ-GT, GLU, TBA, ALB, CHO, TG, LDL, and HDL in six groups, respectively.) OCA: Obeticholic acid; TRIC Acid: 10,12-tricosadiynoic acid. * indicates *p*-value <0.05; ** indicates *p*-value <0.01; *** indicates *p*-value <0.001; **** indicates *p*-value <0.0001; ns indicates *p*-value >0.05.

To investigate the effectiveness of ACOX1-specific inhibitors combined with OCA in treating NASH, wild-type mice were fed a high-fat diet for 14 weeks to induce NAFLD. The high dose of OCA decreased serum TBIL and CHO and increased ALB, but it also significantly reduced serum HDL (Figures 4D, J, F, L, respectively) (*p* < 0.05). Normal doses of OCA were only effective in lowering serum CHO (Figure 4J) (*p* < 0.05). On the other hand, 10,12-Tricosadiynoic acid (an ACOX1-specific inhibitor) significantly reduced serum AST, TG, CHO, and LDL (Figures 4B, I–K, respectively) (*p* < 0.05). Notably, when combined with middle or low-dose OCA, 10,12-Tricosadiynoic acid significantly reduced serum AST, TG, CHO, and LDL, while maintaining normal levels of HDL (Figures 4B, I–L, respectively) (*p* < 0.05). There were no significant differences in serum γ-GT, TBIL, DBIL, ALB, ALP, TBA and HDL among the groups of mice (Figures 4 C-H, L, respectively, *p* > 0.05). These results suggest that ACOX1-specific inhibitors combined with middle or low-dose OCA may be a practical therapeutic approach for NAFLD, with potential benefits in improving transaminases, LDL and steatohepatitis in mice.

**FIGURE 5:**
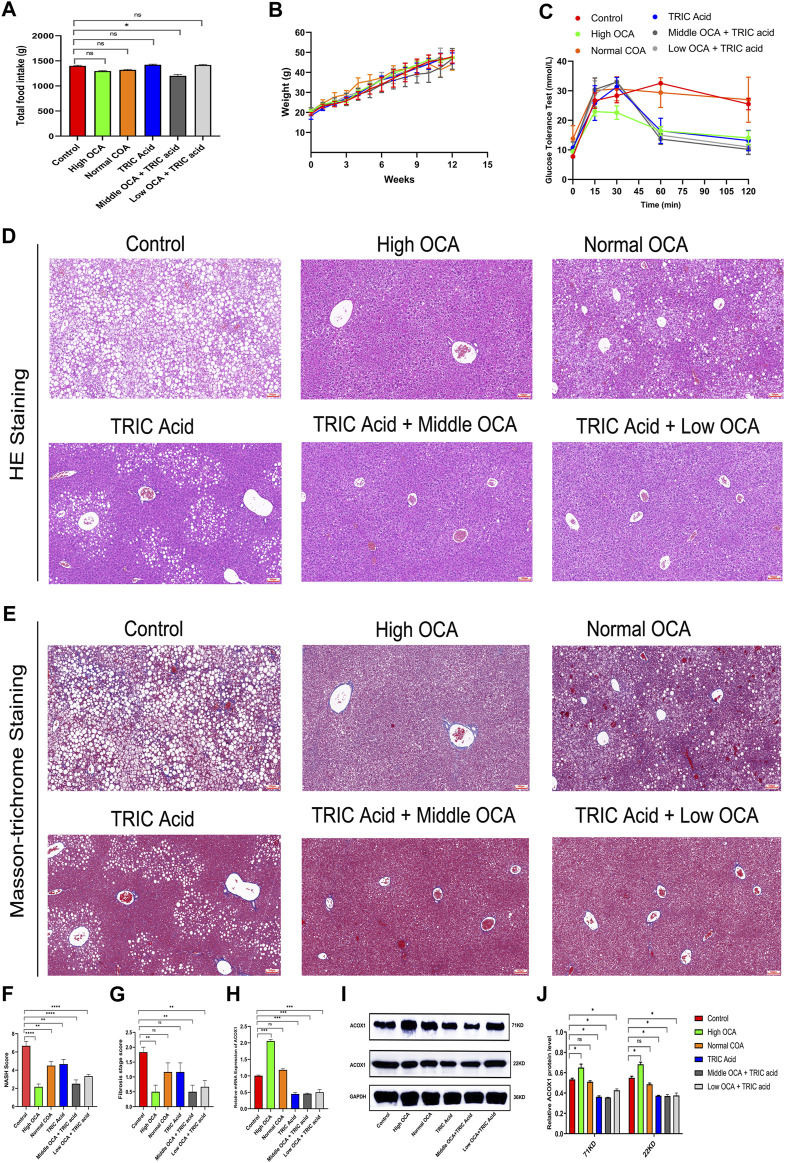
10,12-tricosadiynoic acid combined with middle- or low-dose of obeticholic acid significantly improved steatohepatitis in wild-type mice. (Wild-type mice were fed high-fat diets for 14 weeks to induce nonalcoholic fatty liver disease. **(A–C)** The results of total food intake, weight gain and glucose tolerance in six groups, respectively. **(D,E)** The results of Hematoxylin-eosin staining and Masson’s trichrome staining in six groups, respectively. **(F,G)** The results of NASH score and fibrosis stage scores in six groups, respectively. **(H–J)** The qPCR and Western Blot results about ACOX1 expression in three groups, respectively. OCA: obeticholic acid; TRIC Acid: 10,12-tricosadiynoic acid). OCA: Obeticholic acid; TRIC Acid: 10,12-tricosadiynoic acid. * indicates *p*-value <0.05; ** indicates *p*-value <0.01; *** indicates *p*-value <0.001; **** indicates *p*-value <0.0001; ns indicates *p*-value >0.05.

There were no significant differences in total food intake, body weight gain and glucose tolerance among the six groups of mice (Figures 5A, B) (*p* > 0.05). However, except for the normal-dose OCA group, the other intervention groups had less impaired glucose tolerance than the control group (Figure 5C). Hematoxylin-eosin staining showed that steatohepatitis was significantly reduced, and NASH scores were significantly lower in mice receiving high-dose OCA or 10,12-Tricosadiynoic acid combined with middle or low-dose OCA compared to the control group (Figures 5D, F) (*p* < 0.001). Although the normal dose of OCA or 10,12-Tricosadiynoic acid reduced steatohepatitis, their efficacy was not as good as the other groups (Figures 5D, F) (*p* < 0.05). Compared to the control group, high-dose OCA, 10,12-Tricosadiynoic acid combined with middle- or low-dose OCA reduced liver fibrosis, although the fibrosis scores failed to reach statistical significance (Figures 5E, G) (*p* > 0.05). High doses of OCA significantly upregulated ACOX1 mRNA and protein, but normal doses had little effect (Figures 5H–J) (*p* < 0.05). However, 10,12-Tricosadiynoic acid combined with middle- or low-doses of OCA significantly decreased ACOX1 mRNA and protein expression in mice’s liver (Figures 5H–J) (*p* < 0.05).

Combining ACOX1-specific inhibitors with middle- or low-dose OCA effectively improved liver function and reduced LDL and steatohepatitis in mice, indicating that targeting ACOX1 could be a promising therapeutic strategy for NAFLD when used in conjunction with FXR agonists.

### 3.5 ACOX1-specific inhibitors improve the efficacy and reduce the toxicity of OCA via inhibiting IL-β and α-SMA pathways in NAFLD treatment

**FIGURE 6:**
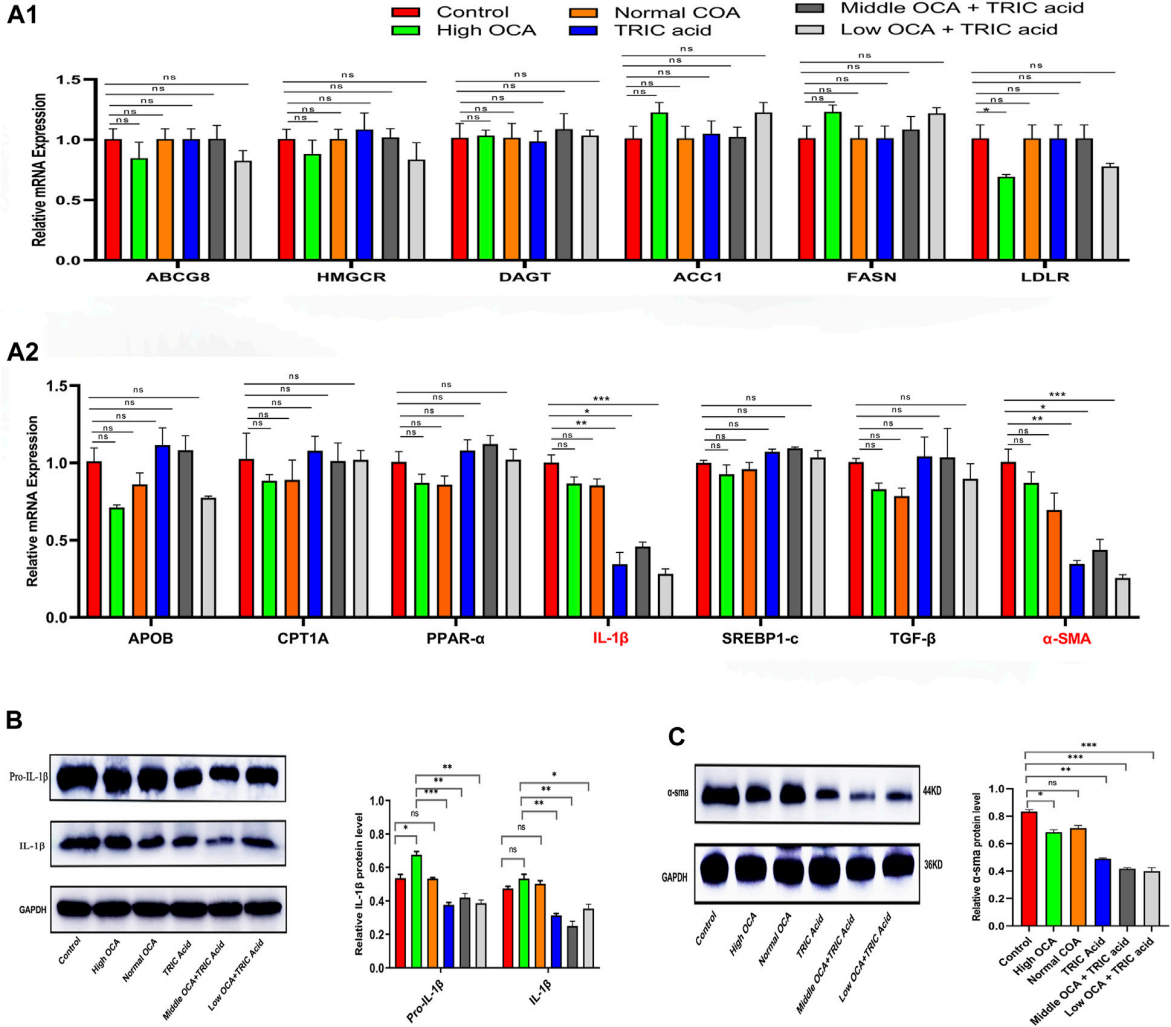
ACOX1-specific inhibitors improve obeticholic acid’s effectiveness and reduce its toxicity in treating non-alcoholic fatty liver disease by inhibiting IL-1β and α-SMA pathways. **(A1,A2)** The qPCR results for ABCG8, HMGCR, DAGT, ACC1, FASN, LDLR, APOB, CPT1A, PPAR-α, IL-1β,SREBP1-c, TGF-β and α-SMA in wild-type mice NAFLD model. **(B,C):** The Western Blot results of IL-1β and α-SMA in wild-type mice NAFLD model and fibrosis models, respectively. OCA: obeticholic acid; TRIC Acid: 10,12-tricosadiynoic acid.) * indicates *p*-value <0.05; ** indicates *p*-value <0.01; *** indicates *p*-value <0.001; **** indicates *p*-value <0.0001; ns indicates *p*-value >0.05.

To investigate the underlying mechanisms, qPCR analysis was performed on genes related to lipid and cholesterol metabolism, inflammation and fibrosis. The results showed that the mRNA expression of most analyzed genes (ABCG8, HMGCR, DAGT, ACC1, FASN, LDLR, APOB, CPT1A, PPAR-α, SREBP1-c, and TGF-β) was not significantly different among the six groups of mice with NAFLD (Figures 6A1, A2) (*p* < 0.05). However, IL-1β and α-SMA mRNA levels were significantly lower in the 10,12-Tricosadiynoic acid group and in the 10,12-Tricosadiynoic acid combined with the middle- or low-doses of the OCA groups (Figures 6A1, A2) (*p* < 0.05). Similarly, the protein expression of IL-1β and α-SMA was significantly downregulated in these three groups compared to the other groups in NAFLD models (Figures 6B, C) (*p* < 0.05). Therefore, the combination of ACOX1-specific inhibitors with middle- or low-dose OCA may improve the therapeutic effects and reduce OCA toxicity due to the reduction of IL-β and α-SMA pathways.

## 4 Discussion

### 4.1 Optimizing OCA therapy: ACOX1-specific inhibitors mitigate lipotoxicity and enhance efficacy in NAFLD treatment

High-dose OCA is an effective treatment for steatohepatitis and fibrosis in NAFLD patients. Still, its excessive activation of FXR can cause lipotoxicity, leading to adverse effects on LDL and HDL levels. These adverse reactions are correlated with the activation of ACOX1. However, inhibition of ACOX1 expression with 10,12-Tricosadiynoic acid in combination with middle- or low-dose OCA significantly improves steatohepatitis and liver fibrosis, with efficacy comparable to high-dose OCA, but without adverse effects on LDL and HDL levels. Furthermore, ACOX1 inhibition also showed inhibitory effects on IL-1β and α-SMA signaling pathways, which are involved in the progression of NAFLD. Therefore, ACOX1-specific inhibitors can potentially reduce toxicity and improve the therapeutic effects of OCA in NAFLD treatment.

### 4.2 Balancing FXR agonists in NAFLD treatment: ACOX1-specific inhibitors as promising allies

FXR is a natural ligand for bile acids, and its agonists have been shown to promote the β-oxidation of fatty acids and inhibit several pathways involved in NAFLD, including bile acid synthesis, hepatic and intestinal recycling of bile acids, lipid synthesis, gluconeogenesis, and fatty acid uptake by hepatocytes (Carr and Reid, 2015; Xi and Li, 2020; Lin et al., 2022b). While FXR agonists hold great promise as a treatment option for NAFLD, their use is limited due to side effects. For example, GW4064, Cilofexor and HEC9671 have been shown to improve steatohepatitis and hepatic fibrosis in animal models, but their side effects limit their use in humans (Younossi et al., 2019; Patel et al., 2020; Cao et al., 2022). OCA, a highly potent semi-synthetic FXR agonist, has been approved by the FDA and the EU for treating primary biliary cholangitis and a phase III clinical study has demonstrated that high-dose OCA improves steatohepatitis and liver fibrosis in NASH patients (Nevens et al., 2016; Younossi et al., 2019). However, high-dose OCA has been associated with adverse effects such as pruritus, elevated serum LDL and decreased HDL, which limit its use in NAFLD patients (Hirschfield et al., 2015; Younossi et al., 2019; Siddiqui et al., 2020). In our previous study, it was shown that the toxicity of high-dose OCA depends on the presence and activation of FXR (Lin et al., 2022a). Furthermore, the present study has demonstrated that FXR causes lipotoxicity by upregulating ACOX1 expression, which is only upregulated by high doses of OCA and not at regular or low doses. Combining ACOX1-specific inhibitors, such as 10,12-tricosadiynoic acid, with middle or low doses of OCA has been shown to effectively improve liver function, steatohepatitis and liver fibrosis in animal models, with comparable efficacy to high doses of OCA, but without the adverse effects on LDL and HDL levels. Therefore, combining ACOX1-specific inhibitors with middle- or low-dose OCA may be a successful therapeutic strategy for NAFLD patients.

### 4.3 ACOX1 as a therapeutic target in NAFLD: unraveling the role of very long-chain fatty acid β-oxidation

ACOX1 is an essential rate-limiting enzyme in the β-oxidation of very long-chain fatty acids in peroxisomes. ACOX1 catalyzes the desaturation of acyl-CoAs to 2-trans-enoyl-CoAs, which produces two copies of Acetyl-CoA and a large amount of hydrogen peroxide. Increased Acetyl-CoA from this pathway promotes adipose autophagy and inhibits mitochondrial fatty acid β-oxidation, which exacerbates the progression of NAFLD. Additionally, the hydrogen peroxide generated by ACOX1 activity increases oxidative stress and can cause cell damage and even cell death (He et al., 2020a; He et al., 2020b). Previous studies have shown that ACOX1 gene liver-specific knockout reduced hepatic steatosis induced by starvation or a high-fat diet in mice and that the ACOX1-specific inhibitor 10,12-tricosadiynoic acid alleviated hepatic steatosis in rats (Zeng et al., 2017; He et al., 2020b). In the present study, inhibition of ACOX1 by 10,12-tricosadiynoic acid improved steatohepatitis and liver fibrosis and significantly reduced liver inflammation and serum LDL levels. These findings suggest that ACOX1 is an effective therapeutic target for NAFLD.

### 4.4 ACOX1 inhibition attenuates liver inflammation and fibrosis: modulating IL-1β and α-SMA as a therapeutic avenue in NAFLD

In addition, this study found that inhibition of ACOX1 led to decreased expression of IL-1β and α-SMA at both mRNA and protein levels. IL-1β is a proinflammatory cytokine that plays a vital role in developing liver hepatitis and fibrosis. It activates Kupffer cells and HSCs, inducing the production of various inflammatory cytokines, chemokines, and ROS, thereby promoting liver inflammation (Tilg and Moschen, 2010). Furthermore, IL-1β stimulates the proliferation and differentiation of HSCs into myofibroblasts, contributing to the deposition of extracellular matrix and fibrosis development. Additionally, IL-1β induces the expression of fibrogenic factors, such as TGF-β, CTGF and PDGF, which further promote liver fibrosis (Kisseleva and Brenner, 2008). Overall, IL-1β is a crucial player in the pathogenesis of liver inflammation and fibrosis and is a potential therapeutic target for anti-inflammatory and anti-fibrotic therapies (Gabay et al., 2010; Tacke and Trautwein, 2015). Increased IL-1β secretion upregulates TIMP-1, inhibiting extracellular matrix degradation and fibroblast apoptosis and aggravating liver fibrosis. Inhibition of IL-1β has been shown to improve steatohepatitis and liver fibrosis in animal models (Du et al., 2019; Vergis et al., 2021). In the present study, downregulation of IL-1β expression significantly improved steatohepatitis and fibrosis in mice.

Alpha-smooth muscle actin (α-SMA) is a cytoskeletal protein commonly expressed in activated HSCs, the main cells involved in liver fibrosis (Tsuchida and Friedman, 2017). HSCs play a key role in extracellular matrix production, tissue remodeling and fibrosis progression in the liver (Kisseleva and Brenner, 2008). Activation of HSCs leads to the upregulation of α-SMA expression, which promotes the contraction and migration of HSCs to the injury site. Additionally, α-SMA expression stimulates the secretion of profibrogenic cytokines, such as TGF-β, further exacerbating liver fibrosis (Friedman, 2008; Rockey et al., 2019). α-SMA deficiency has been shown to improve liver fibrosis (Rockey et al., 2019). In summary, α-SMA promotes the development of hepatitis and liver fibrosis by promoting HSC activation, migration and extracellular matrix production. In the present study, the downregulation of α-SMA expression significantly improved steatohepatitis and fibrosis in mice.

### 4.5 ACOX1 inhibition enhances NAFLD treatment: mechanisms, synergy with OCA, and therapeutic potential

**FIGURE 7:**
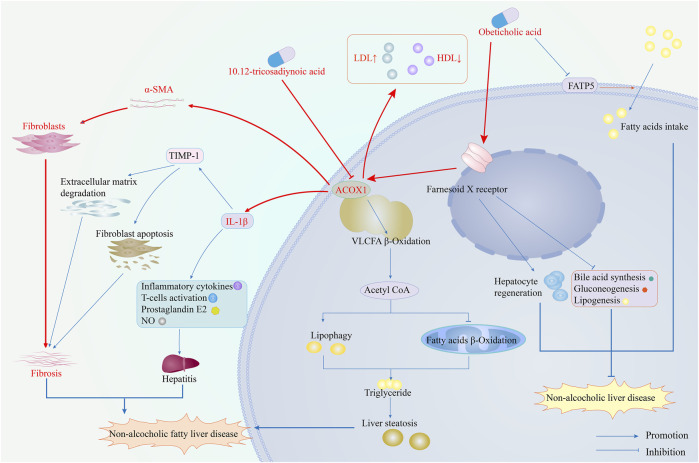
The mechanism of ACOX1-specific inhibitors improves obeticholic acid’s effectiveness and reduces its toxicity in treating non-alcoholic fatty liver disease. (Note: The red arrows represent the results of this study, while the blue ones indicate supporting evidence from the literature).

Inhibiting ACOX1 expression effectively improves steatohepatitis and liver fibrosis and inhibits the IL-1β and α-SMA pathways. ACOX1-specific inhibitors, in combination with OCA, synergistically treat NAFLD by improving efficacy and reducing toxicity. According to existing literature, ACOX1 inhibitors achieve this by suppressing ACOX1 expression, reducing lipid autophagy, and increasing fatty acid β-oxidation, thereby decreasing hepatic triglyceride levels. Ultimately, this leads to improved liver function and lower serum LDL levels (He et al., 2020a). Figure 7 demonstrates the mechanism of action of ACOX1-specific inhibitors and their ability to improve OCA efficacy while reducing toxicity in NAFLD treatment.

The efficacy and safety of ACOX1-specific inhibitors in combination with OCA have not been evaluated in clinical trials. Clinical validation is essential to confirm the therapeutic potential of the proposed approach in real-world settings.

#### 4.5.1 Limitations and future directions

The limitation of this study is the absence of ACOX1 promoter mutation experiments and the determination of the mechanism by which FXR regulates ACOX1 expression. In future studies, we will investigate this aspect further. We will also explore the long-term safety and efficacy of the combined use of ACOX1-specific inhibitors with OCA and the specificity and selectivity of ACOX1 inhibitors. Longitudinal studies would provide valuable insights into the durability and sustainability of the treatment effects over time.

## 5 Conclusion

Combining ACOX1-specific inhibitors with low-dose obeticholic acid effectively treats high-fat diet-induced hepatic steatosis and reduces serum LDL. This approach enhances the therapeutic effects of obeticholic acid and reduces its lipotoxicity by inhibiting the IL-1β and α-SMA pathways.

## Data Availability

Publicly available datasets were analyzed in this study. This data can be found here: https://www.ncbi.nlm.nih.gov/sra/PRJNA1086042 (NCBI, accession number: PRJNA1086042).

## Supplementary material

SUPPLEMENTARY FIGURE S1 The construction of the vector.

SUPPLEMENTARY FIGURE S2 ACOX1 is one of the most FXR-driven downstream genes regulated by FXR. (RNA-seq data was obtained from the livers of wild-type mice and FXR^−/−^ mice fed a high-fat diet for 14 weeks. **(A)** 15988 genes were co-expressed in WT and FXR^−/−^ mice. **(B)** 2035 genes were down-regulated and 1451 genes were up-regulated in FXR^−/−^ mice. **(C)** KEGG Pathways analysis identified 142 genes related to lipid metabolism that were differentially expressed between WT mice and FXR^−/−^ mice. **(D)** The results of KEGG models analysis about these 142 genes related to lipid metabolism. **(D)** Mutual gene network analysis identified ACOX1 as the central driver among these 142 differential genes of lipid metabolism. **(F)** The results of KEGG network analysis of ACOX1-related differential genes. **(G,H)** ACOX1 expression is down-regulated in the liver of FXR^−/−^ mice.
