# Supplementary material for: Inhibition of ACOX1 enhances the therapeutic efficacy of obeticholic acid in treating non-alcoholic fatty liver disease and mitigates its lipotoxicity
Source: Front Pharmacol. 2024 Mar 26;15:1366479. doi: 10.3389/fphar.2024.1366479 (PMC11003388; doi:10.3389/fphar.2024.1366479)
Supplement: Supplementary file 1 [file DataSheet1.docx]

**Supplementary Table 1: siRNA sequence for interfering plasmid FXR**

| **Marker** | **Gene** | **Gene ID** | **Target Seq** |
| --- | --- | --- | --- |
| FXR | FXR | NM_001206993.2 | GCCTCAGGAAATAACAAATAA |
| NC | NC | NA | CCTAAGGTTAAGTCGCCCTCG |

**Supplementary Table 2:** **The detailed information the different kits used for Metabolic plasma analysis.**

| **Name** | **Catalog** | **lot number** | **company** |
| --- | --- | --- | --- |
| ALB | DL13A1 | 22011305 | Guangzhou Donglin Biotechnology |
| ALT | DL06A1 | 22010601 | Guangzhou Donglin Biotechnology |
| AST | DL23A1 | 22012301 | Guangzhou Donglin Biotechnology |
| GGT | DL04A1 | 22030401 | Guangzhou Donglin Biotechnology |
| TBIL | DL22A1 | 22032201 | Guangzhou Donglin Biotechnology |
| DBIL | DL21A1 | 22032205 | Guangzhou Donglin Biotechnology |
| TBA | E003-2-1 | 20220701 | Guangzhou Donglin Biotechnology |
| Cr | DL13A1 | 22071305 | Guangzhou Donglin Biotechnology |
| CHO | DL23A1 | 22032301 | Guangzhou Donglin Biotechnology |
| TG | DL19A1 | 22031901 | Guangzhou Donglin Biotechnology |
| LDL | DL18A1 | 22041801 | Guangzhou Donglin Biotechnology |
| HDL | DL18A2 | 22031801 | Guangzhou Donglin Biotechnology |
| GLU | DL23A2 | 22012301 | Guangzhou Donglin Biotechnology |

Note：ALT: alanine aminotransferase; AST: aspartate aminotransferase; TBIL: total bilirubin; DBIL: direct Bilirubing; ALB: Albumin; CHO: total cholesterol; TG: triglyceride; HDL: high-density lipoprotein; LDL: low-density lipoprotein; GLU: glucose, r-GT: γ-glutamyltransferase; TBA: total bile acid.

Supplementary Table 3: Primers used for RT-qPCR

| Genes | primers（5’-3’） | Primer sequences |
| --- | --- | --- |
| GAPDH (Human) | Forward primer | AACAGCCTCAAGATCATCAGCAAT |
|  | Reverse primer | TTCCACGATACCAAAGTTGTCA |
| GAPDH (Mouse) | Forward primer | GCCTCCTCCAATTCAACCCT |
|  | Reverse primer | CCCAATACGGCCAAATCCGT |
| ACOX1 (Human) | Forward primer | GCACCATTGCCATCCGATAC |
|  | Reverse primer | CTGTGGTTCTGGTTCACCTGG |
| ACOX1 (Mouse) | Forward primer | TGAAATATGCCCAGGTGAAGC |
|  | Reverse primer | AGACTCTGAGCTGCACTTCC |
| ABCG8 (Mouse) | Forward primer | CTAGGGGGCGACAAGAGAAG |
|  | Reverse primer | TCACGTCGAGTAGTGAGGCT |
| HMGCR (Mouse) | Forward primer | TGCCTGGATGGGAAGGAGTA |
|  | Reverse primer | GCACCTCCACCAAGGCTTAT |
| DAGT (Mouse) | Forward primer | GGTAGTGGGCCCAAGGTAGA |
|  | Reverse primer | TGCAGACGATGGCACCTCAG |
| ACC1 (Mouse) | Forward primer | GACTTCCCGTTGGCATTTGG |
|  | Reverse primer | CGGGTGGTCTTTCAACCAGA |
| FASN (Mouse) | Forward primer | GCAGCTGTTGGTTTGTCCTG |
|  | Reverse primer | ATTCACTGCAGCCTGAGGTC |
| LDLR (Mouse) | Forward primer | CCAATCGACTCACGGGTTCA |
|  | Reverse primer | ACAGTGTCGACTTCTCTAGGC |
| APOB (Mouse) | Forward primer | CTACTTCCACCCACAGTCCC |
|  | Reverse primer | AGGGAGCCTAGCAATCTGGA |
| CPT1a (Mouse) | Forward primer | GGGCCATCTGTGGGAGTATG |
|  | Reverse primer | ACTGTAGCCTGGTGGGTTTG |
| PPARa (Mouse) | Forward primer | GTGCAGCCTCAGCCAAGTT |
|  | Reverse primer | TGGGGAGAGAGGACAGATGG |
| SREBP1c (Mouse) | Forward primer | TCTTGACCGACATCGAAGACAT |
|  | Reverse primer | CCTGTGTCTCCTGTCTCACC |
| IL-1β (Mouse) | Forward primer | TGCCACCTTTTGACAGTGATG |
|  | Reverse primer | TGATGTGCTGCTGCGAGATT |
| α-SMA (Mouse) | Forward primer | AGCCATCTTTCATTGGGATGG |
|  | Reverse primer | CCCCTGACAGGACGTTGTTA |
| TGF-1β(Mouse) | Forward primer | GCAACAATTCCTGGCGATACC |
|  | Reverse primer | AAAGCCCTCAATTTCCCCCTCC |

**Supplementary figure 1:**

**
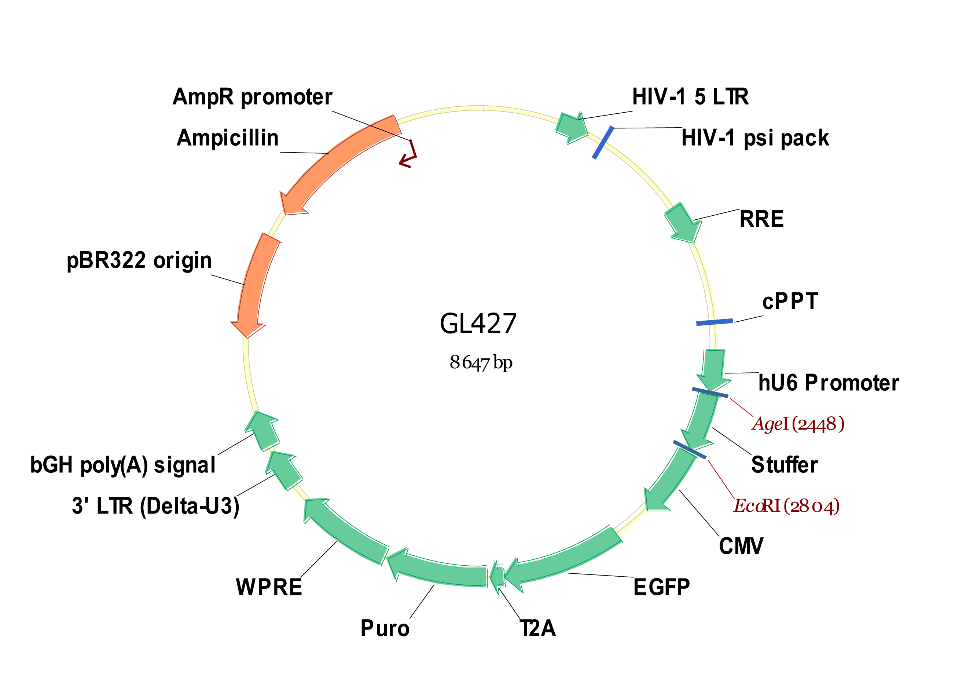
**

**Supplementary figure
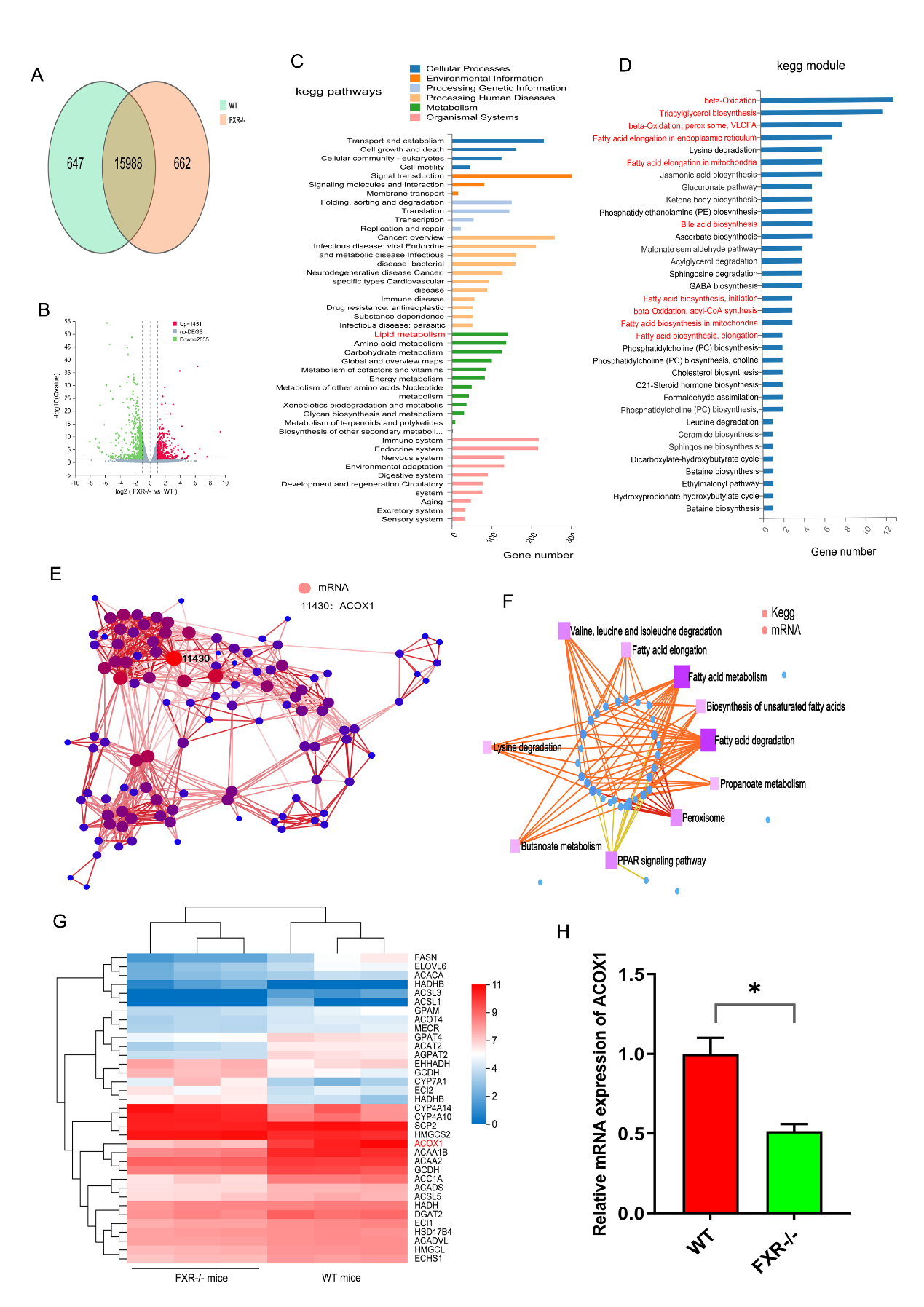
2:**
